# Post term pregnancy in a non-communicating rudimentary horn of a unicornuate uterus

**DOI:** 10.1186/s13104-016-2013-7

**Published:** 2016-04-11

**Authors:** Vitalis Fambombi Feteh, Christian Akem Dimala, Tsi Njim, Bananga Fuka

**Affiliations:** Mboppi Baptist Hospital Douala, P.O Box 15161, Akwa/Douala, Douala, Cameroon; Health and Human Development (2HD) Research Group, Douala, Cameroon; Faculty of Epidemiology and Population Health, London School of Hygiene and Tropical Medicine, London, UK; Regional Hospital Bamenda, North West Region, Bamenda, Cameroon

**Keywords:** Rudimentary horn pregnancy, Unicornuate uterus, Stillbirth, Post term, Cameroon

## Abstract

**Background:**

Pregnancy in a rudimentary horn of a unicornuate uterus is rare in obstetrics and when it occurs, it seldom progresses to term as ruptures frequently occur before the third trimester.

**Case history:**

A 29 year old female, presented at 42 weeks 5 days complaining of absent foetal movements, with results of a self-prescribed ultrasound scan showing an “abdominal pregnancy with foetal demise”. She was haemodynamically stable and there were no foetal heart tones. At laparotomy, a non-communicating rudimentary horn pregnancy (RHP) was discovered. The right horn and tube were resected, with delivery of a post term female stillbirth. There were no postoperative complications.

**Conclusion:**

Rudimentary horn pregnancies are difficult to diagnose when advanced; especially in low-resource settings with suboptimal antenatal care. Maternal and foetal outcomes in RHPs are usually poor; RHPs should therefore be suspected in pregnancies with atypical ultrasonographic features and more investigations done to confirm the diagnosis in order to reduce the associated morbidity and mortality.

## Background

Rudimentary horn pregnancy (RHP) is a very rare obstetric entity with a reported incidence from 1/100,000 to 1/140,000 pregnancies [1]. Hitherto, it was unreported in Cameroon [2]. Early diagnosis and management is important, though challenging especially in low-resource settings where diagnostic modalities like: ultrasonography, hysterosalpingography, hysteroscopy, laparoscopy, and MRI [3] are widely unavailable or their use inadequate.

The clinical course of this condition is usually characterized by a high rate of uterine rupture [2] with 85 % of pregnancies in a non-communicating rudimentary horn typically rupturing before the third trimester. These pregnancies rarely reach term, and when they do, foetal outcome is often poor, with a reported 6 % survival rate [1]. This situation is particularly compounded by suboptimal antenatal care (ANC). Current literature emphasizes the importance of state-of-the-art sonographic prowess in its diagnoses and timely intervention to optimize outcome. We report a first ever case of a rare post-term still birth from a rudimentary horn pregnancy, initially missed on ultrasound scan (USS) at 21 weeks then misdiagnosed twice in the third trimester as an abdominal pregnancy.

## Case history

A 29-year-old female school teacher, G_2_P_0_010, blood group O rhesus positive presented at our antenatal clinic for the first time with the result of her obstetric USS at 42 weeks 5 days [dated from a reliable last normal menstrual period (LNMP)].

Antenatal care during pregnancy was inadequate and irregular with her first ANC consultation done at a peripheral health centre at 9 weeks 5 days. The evolution of the pregnancy was unremarkable and she had her first USS at 21 weeks, which reported: “a single viable intrauterine pregnancy” with foetal parameters corresponding to a 20 week gestation. At 41 weeks 3 days, results of a self-prescribed USS (done at another commercial imaging centre) reported: “a single viable abdominal pregnancy with a breech presentation and oligohydramnios”. The patient was not advised to seek an obstetric consult. Ten days later, absence of foetal movements for over 24 h, prompted the patient to request another USS which revealed “an abdominal pregnancy with foetal demise”. She subsequently consulted at our hospital for appropriate management. Upon presentation, she reported the absence of foetal movements for over 36 h, there was no abdominal pain nor fever, she denied any loss of show, vagina bleeding, or gush of fluid. She was conscious and well oriented with pink conjunctivae, a blood pressure of 118/72 mm Hg; a pulse of 66 beats per minute and a temperature of 36.9 °C. Obstetric examination revealed a ‘fundal height’ of 35 cm with an indeterminate foetal lie. She had normal external genitalia and the vagina and cervix appeared macroscopically normal. The cervix was posterior, soft, 1.5 cm long and cervical os was closed. Repeat USS reported “an empty uterus, with foetal demise on the right of the uterus”. Her haemoglobin level was 12.4 g/dL and serum creatinine value was 1.0 mg/dL. She was admitted and prepared for laparotomy with indication for evacuation of an abdominal pregnancy.

### Laparotomy was done under general anaesthesia with a midline infra umbilical incision

**Fig. 1:**
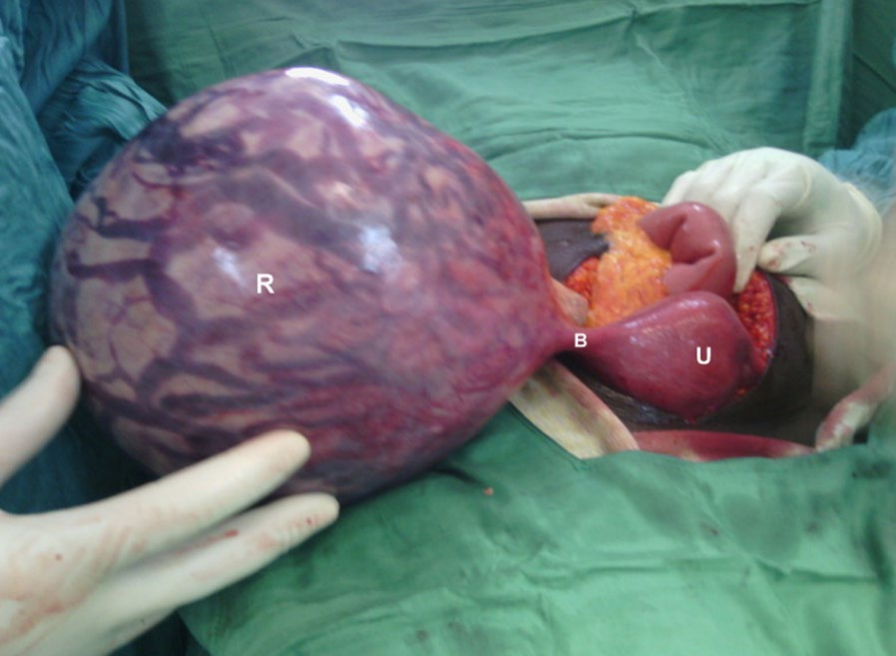
Showing non communicating rudimentary horn pregnancy (*R*) connected to unicornuate uterus (*U*) through a fibrous band (*B*)

Intraoperatively, a right-sided rudimentary horn pregnancy was discovered, which was connected to the main uterine cavity with a thick 1.5 cm fibrous band. The gravid horn was attached directly to the fimbriated end of the right tube. There was no communication of the gravid horn with the cervix nor with the normal horn (Fig. 1).

Excision of the gravid rudimentary horn with right salpingectomy was done en bloc. Haemostasis was assured. Dissection of the gestational sac revealed a non-viable 2300 g, female neonate, APGAR 0/10 with post-term features (abundant scalp hair; no lanugo; desquamating skin; diminished vernix caseosa, long finger nails and meconium stains) and no obvious external congenital malformations.

Postoperative stay was uneventful and patient was discharged on day 5, with advice to seek early and qualified medical care in subsequent pregnancies. An abdominal scan and intravenous urography at 8 weeks postpartum revealed no associated urological anomalies.

## Discussion

Developmental anomalies of the female urogenital tract are not uncommon. The exact prevalence of these anomalies is difficult to determine due to its clinical subtlety [4]. The incidence of defective fusion of the paramesonephric (Müllerian) ducts is estimated to be 0.1–3 % in unselected populations [5], but increases from 2 to 8 % amongst infertile women and 5–30 % of women with a history of miscarriages [6].

*Uterus bicornis unicollis* (or unicornuate uterus) with one rudimentary horn is an anomaly that results from unilateral hypoplasia of the uterine ducts. It is classified as type U4a, according to the European Society of Human Reproduction and Embryology (ESHRE) and the European Society for Gynaecological Endoscopy (ESGE) [ESHRE/ESGE] system [7]. It may present a rudimentary horn without or with a functional endometrium, the latter being more prone to obstetric complications. It is postulated that pregnancy can occur in a non-communicating rudimentary horn via trans-peritoneal migration of spermatozoa or fertilized ovum from contralateral side, then implantation in the horn [4].

Rudimentary horn pregnancies are rare, difficult to diagnose and seldom progress to term as evolution is usually marked by uterine rupture in most cases. However, there has been a reported case of live term delivery from a RHP in rural Nigeria [8].

Only about 26 % of RHPs are diagnosed pre-operatively [9]. In 74 % of cases the diagnosis is often missed by ultrasonography (the most widely used investigation during pregnancy), and sensitivity of sonography decreases as pregnancy advances beyond first trimester. It can be missed in inexperienced hands or with low quality instruments. Tubal pregnancy, cornual pregnancy, intrauterine pregnancy, and abdominal pregnancy are common sonographic misdiagnoses [10]. Magnetic resonance imaging may be needed for confirmation [11] but this investigation is widely unavailable in Cameroon and where present, it is expensive. In our case, a RHP was misdiagnosed twice as an abdominal pregnancy by obstetric ultrasounds done in the second and third trimesters, highlighting the potential importance of first trimester USS [11]. However, these misdiagnoses could be explained, as abdominal pregnancies are about 10 more common than RHPs [9]. This pregnancy was dated using a reliable last normal menstrual period and was well-nigh confirmed by a second trimester ultrasound.

A RHP can also be suspected on early clinical pelvic exam, wherein a mass extending outside the uterine angle can sometimes be felt on bimanual examination (*Baart de la Faille’s sign*) or displacement of the fundus to the contralateral side with rotation of the uterus and elevation of the affected horn (*Ruge Simon syndrome*) [12]. Using ultrasonography, Tsafrir et al. [11] outlined the following set of criteria for early diagnosis of RHPs: (1) a pseudo pattern of asymmetrical bicornuate uterus; (2) absent visual continuity tissue surrounding the gestation sac and the uterine cervix; (3) presence of myometrial tissue surrounding the gestation sac; (4) hypervascularization typical of placenta accreta [7]. Withal, these criteria may be important only in the first trimester.

Congenital uterine anomalies may lead to infertility; recurrent first-trimester pregnancy loss and other obstetrical complications like increased risk of preterm birth; preterm premature rupture of membranes; breech presentation; cesarean section; placenta previa; placental abruption and intrauterine growth retardation (IUGR) [4]. Our patient had one spontaneous abortion in the past for which she was uninvestigated hence the uterine anomaly remained undiagnosed. This emphasizes the need for patient-specific ANC consultations. Also the foetus had intrauterine growth restriction, partly explained by the restricted space in the rudimentary horn and poor placentation.

Furthermore, in our case, the obstetrical outcome was somewhat compounded by: sub-optimal ANC; the lack of early referral; the phenomenon of self-prescribed investigation and the absence of qualified sonographers in the numerous commercial imaging centres in Cameroon.

This patient had a resection of the rudimentary horn and an ipsilateral total salpingectomy- the recommended treatment option- to prevent future ipsilateral ectopic pregnancies. Her prognosis for future pregnancies has been reduced. She has no increased risk of uterine scar rupture in subsequent pregnancies. There were also no associated urinary tract malformations—a common coincidence with genital tract malformations.

## Conclusion

Rudimentary horn pregnancy is a rare clinical entity whose diagnosis is still difficult especially in resource-poor settings, where ANC remains suboptimal and access to thorough sonographic imaging is widely unavailable; with resulting poor maternal and foetal outcomes. In order to increase the probability of early diagnosis and improve the prognosis in resource-limited settings, this entity should be suspected by clinicians in some pregnancies with atypical ultrasonography findings and more robust investigations implored to confirm diagnosis.

## Abbreviations

ANC: antenatal care
ESGE: European Society for Gynaecological Endoscopy
ESHRE: European Society of Human Reproduction and Embryology
IUGR: intrauterine growth restriction
LNMP: last normal menstrual period
USS: ultrasound scan
RHP: rudimentary horn pregnancy
